# (*Z*)-3-Diethyl­amino-6-({2-[(*E*)-4-(diethyl­amino)-2-hy­droxy­benzyl­idene­amino]-4,5-dimethyl­phen­yl}amino­methyl­idene)cyclo­hexa-2,4-dienone–5,5′-bis­(diethyl­amino)-2,2′-[4,5-dimethyl-*o*-phenyl­enebis(nitrilo­methyl­idyne)]diphenol

**DOI:** 10.1107/S1600536810045290

**Published:** 2010-11-10

**Authors:** Hadi Kargar, Reza Kia, Muhammad Nawaz Tahir

**Affiliations:** a Department of Chemistry, School of Science, Payame Noor University (PNU), Ardakan, Yazd, Iran; b Department of Chemistry, Science and Research Branch, Islamic Azad University, Tehran, Iran; c X-ray Crystallography Lab., Plasma Physics Research Center, Science and Research Branch, Islamic Azad University, Tehran, Iran; d Department of Physics, University of Sargodha, Punjab, Pakistan

## Abstract

The asymmetric unit of the title Schiff base compound, C_30_H_38_N_4_O_2_, comprises two crystallographically independent mol­ecules, *A* and *B*. The structure is non-merohedrally twinned with a refined BASF ratio of 0.219 (6):0.701 (6). Mol­ecule *B* shows both phenol–imine and keto–amine tautomeric forms in a single structure. The dihedral angles between the central ring and the two outer rings are 5.9 (3) and 48.4 (3)° in mol­ecule *A*, and 48.3 (3) and 6.9 (3)° in mol­ecule *B*. Strong intra­molecular O—H⋯N and N—H⋯O hydrogen bonds generate *S*(6) ring motifs. The crystal structure is further stabilized by inter­molecular C—H⋯O, C—H⋯π and π–π inter­actions [centroid–centroid distances = 3.870 (4)–3.871 (4) Å].

## Related literature

For standard values of bond lengths, see: Allen *et al.* (1987 ▶). For details of hydrogen-bond motifs, see: Bernstein *et al.* (1995 ▶). For related structures, see: Kargar *et al.* (2009 ▶, 2010*a* ▶,*b* ▶).
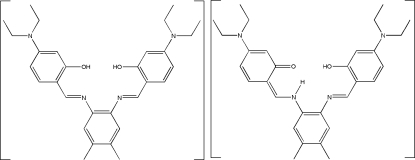


## Experimental

### 

#### Crystal data

C_30_H_38_N_4_O_2_
*M* *_r_* = 486.64
Triclinic, 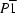

*a* = 11.4430 (12) Å
*b* = 12.0251 (12) Å
*c* = 22.171 (2) Å
α = 88.241 (6)°
β = 89.370 (7)°
γ = 65.207 (6)°
*V* = 2768.2 (5) Å^3^
*Z* = 4
Mo *K*α radiation
μ = 0.07 mm^−1^
*T* = 296 K
0.24 × 0.19 × 0.11 mm

#### Data collection

Bruker SMART APEXII CCD area-detector diffractometer
Absorption correction: multi-scan (*SADABS*; Bruker, 2005 ▶) *T*_min_ = 0.983, *T*_max_ = 0.992
44348 measured reflections
9655 independent reflections
4485 reflections with *I* > 2σ(*I*)
*R*_int_ = 0.095

#### Refinement

*R*[*F* ^2^ > 2σ(*F* ^2^)] = 0.106
*wR*(*F* ^2^) = 0.320
*S* = 1.06
9655 reflections
662 parameters
H-atom parameters constrained
Δρ_max_ = 0.32 e Å^−3^
Δρ_min_ = −0.40 e Å^−3^

### 

Data collection: *APEX2* (Bruker, 2005 ▶); cell refinement: *SAINT* (Bruker, 2005 ▶); data reduction: *SAINT*; program(s) used to solve structure: *SHELXTL* (Sheldrick, 2008 ▶); program(s) used to refine structure: *SHELXTL*; molecular graphics: *SHELXTL*; software used to prepare material for publication: *SHELXTL* and *PLATON* (Spek, 2009 ▶).

## Supplementary Material

Crystal structure: contains datablocks global, I. DOI: 10.1107/S1600536810045290/hg2737sup1.cif

Structure factors: contains datablocks I. DOI: 10.1107/S1600536810045290/hg2737Isup2.hkl

Additional supplementary materials:  crystallographic information; 3D view; checkCIF report

## Figures and Tables

**Table 1:** Hydrogen-bond geometry (Å, °)

*D*—H⋯*A*	*D*—H	H⋯*A*	*D*⋯*A*	*D*—H⋯*A*
O1—H1⋯N1	0.91	1.68	2.553 (7)	160
O2—H2⋯N2	0.90	1.79	2.613 (8)	150
O3—H3⋯N5	0.90	1.85	2.627 (8)	143
N6—H6⋯O4	0.85	1.74	2.562 (7)	162
C7—H7*A*⋯O4	0.93	2.50	3.360 (9)	153
C46—H46*A*⋯O1^i^	0.93	2.54	3.373 (9)	149

Symmetry code: (i) 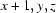.

**Fig. 1.**
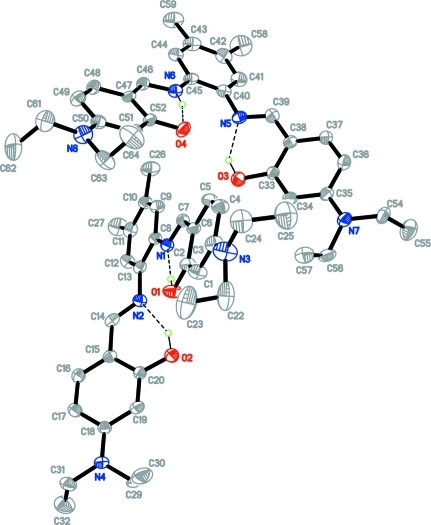
The asymmetric unit of the title compound, showing 30% probability displacement ellipsoids and the atomic numbering. Intramolecular hydrogen bonds are drawn as dashed lines. The H atoms omitted for clarity except those involving in the hydrogen bonds.

**Fig. 2.**
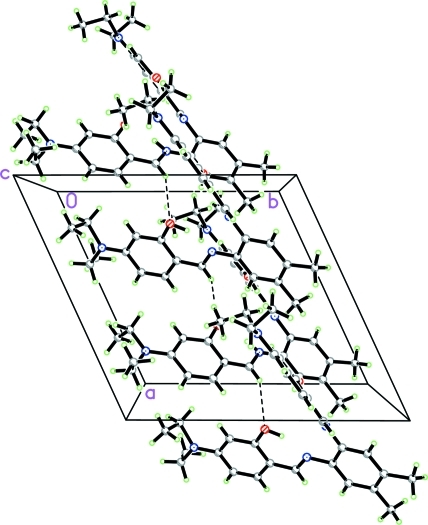
The crystal packing of the title compound viewed down the c-axis, shows linking of molecules along the a-axis through C—H···O hydrogen bonds. Tha dashed lines show interactions.

## supplementary crystallographic information

### Comment

Schiff base ligands are one of the most prevalent systems in coordination chemistry. As part of a general study of tetradenate Schiff bases (Kargar *et al.,* 2009; Kargar *et al.*, 2010**a*,b*), we have determined the crystal structure of the title compound.

The asymmetric unit of the title Schiff base compound, Fig. 1, comprises two crystallographically independent molecules, A and B which is non-merohedrally twinned with a refined BASF ratio of 0.219 (6)/0.701 (6). Molecule B shows both phenol-imine and keto-amine tautomeric form in a single structure. The dihedral angles between the central phenyl ring with the two outer phenyl rings are 5.9 (3) and 48.4 (3)° in molecule A and 48.3 (3) and 6.9 (3)° in molecule B, respectively. Strong intramolecular O—H···N and N—H···O hydrogen bonds generate *S(6)* ring motifs. The crystal structure is further stabilized by the intermolecular C—H···π  and π-π interactions [Cg1···Cg1^i^ = 3.870 (4)Å, (i) -x, 1 - y, 1 - z; Cg2···Cg2^ii^ = 3.871 (4)Å, (ii) 1 - x, 1 - y, -z; Cg1 and Cg2 are the centroids of C15–C20 and C33–C38 benzene rings].

### Experimental

The title compound was synthesized by adding 4-*N*-diethylamino- salicylaldehyde (4 mmol) to a solution of 4,5-dimethyl-1,2-phenylenediamine (2 mmol) in ethanol (20 ml). The mixture was refluxed with stirring for half an hour. The resultant yellow solution was filtered. Yellow single crystals of the title compound suitable for *X*-ray structure determination were recrystallized from ethanol by slow evaporation of the solvents at room temperature over several days. The quality of the crystal was not optimal and it was weakly diffracting. Although recrystallization was attempted repeatedly, we tried three data collections but no better data than this one was obtained.

### Refinement

H atoms of the hydroxy and amino groups were located in a difference Fourier map. They first restrained to 0.90 (1)Å [OH] and 0.85 (1)Å [NH] and then constrained to refine with the parent atoms with U_iso_(H) = 1.5 U_eq_(O) and U_iso_(H) = 1.2 U_eq_(N), see Table 1. The remaining H atoms were positioned geometrically with C-H = 0.93-0.97 Å and included in a riding model approximation with U_iso_ (H) = 1.2 or 1.5 U_eq_ (C). A rotating group model was used for the methyl groups.

### Crystal data

**Table:**

C_30_H_38_N_4_O_2_	*Z* = 4
*M**_r_* = 486.64	*F*(000) = 1048
Triclinic, *P*1	*D*_x_ = 1.168 Mg m^−^^3^
Hall symbol: -P 1	Mo *K*α radiation, λ = 0.71073 Å
*a* = 11.4430 (12) Å	Cell parameters from 2502 reflections
*b* = 12.0251 (12) Å	θ = 2.5–30.5°
*c* = 22.171 (2) Å	µ = 0.07 mm^−^^1^
α = 88.241 (6)°	*T* = 296 K
β = 89.370 (7)°	Block, yellow
γ = 65.207 (6)°	0.24 × 0.19 × 0.11 mm
*V* = 2768.2 (5) Å^3^	

### Data collection

**Table:**

Bruker SMART APEXII CCD area-detector diffractometer	9655 independent reflections
Radiation source: fine-focus sealed tube	4485 reflections with *I* > 2σ(*I*)
graphite	*R*_int_ = 0.095
φ and ω scans	θ_max_ = 25.0°, θ_min_ = 0.9°
Absorption correction: multi-scan (*SADABS*; Bruker, 2005)	*h* = −13→13
*T*_min_ = 0.983, *T*_max_ = 0.992	*k* = −14→14
44348 measured reflections	*l* = −1→26

### Refinement

**Table:**

Refinement on *F*^2^	Primary atom site location: structure-invariant direct methods
Least-squares matrix: full	Secondary atom site location: difference Fourier map
*R*[*F*^2^ > 2σ(*F*^2^)] = 0.106	Hydrogen site location: inferred from neighbouring sites
*wR*(*F*^2^) = 0.320	H-atom parameters constrained
*S* = 1.06	*w* = 1/[σ^2^(*F*_o_^2^) + (0.1053*P*)^2^ + 5.0339*P*] where *P* = (*F*_o_^2^ + 2*F*_c_^2^)/3
9655 reflections	(Δ/σ)_max_ < 0.001
662 parameters	Δρ_max_ = 0.32 e Å^−^^3^
0 restraints	Δρ_min_ = −0.40 e Å^−^^3^

### Special details

**Table:**

Geometry. All esds (except the esd in the dihedral angle between two l.s. planes) are estimated using the full covariance matrix. The cell esds are taken into account individually in the estimation of esds in distances, angles and torsion angles; correlations between esds in cell parameters are only used when they are defined by crystal symmetry. An approximate (isotropic) treatment of cell esds is used for estimating esds involving l.s. planes.
Refinement. Refinement of F^2^ against ALL reflections. The weighted R-factor wR and goodness of fit S are based on F^2^, conventional R-factors R are based on F, with F set to zero for negative F^2^. The threshold expression of F^2^ > 2sigma(F^2^) is used only for calculating R-factors(gt) etc. and is not relevant to the choice of reflections for refinement. R-factors based on F^2^ are statistically about twice as large as those based on F, and R- factors based on ALL data will be even larger.

### Fractional atomic coordinates and isotropic or equivalent isotropic displacement parameters (Å^2^)

**Table:**

	*x*	*y*	*z*	*U*_iso_*/*U*_eq_	
O1	0.1771 (4)	0.4531 (4)	0.2969 (2)	0.0672 (14)	
H1	0.2090	0.5103	0.2982	0.101*	
O2	−0.0627 (4)	0.6876 (4)	0.34677 (19)	0.0623 (12)	
H2	0.0112	0.6973	0.3428	0.093*	
O3	0.4368 (4)	0.6831 (4)	0.15051 (19)	0.0657 (13)	
H3	0.5101	0.6915	0.1571	0.099*	
O4	0.6807 (4)	0.4524 (4)	0.2030 (2)	0.0628 (13)	
N1	0.3133 (5)	0.5755 (4)	0.2929 (2)	0.0443 (12)	
N2	0.1449 (5)	0.7137 (4)	0.3758 (2)	0.0473 (13)	
N3	0.2933 (7)	0.1058 (6)	0.1759 (3)	0.090 (2)	
N4	−0.3082 (5)	0.6070 (5)	0.5023 (2)	0.0609 (15)	
N5	0.6399 (5)	0.7194 (4)	0.1182 (2)	0.0510 (13)	
N6	0.8132 (5)	0.5792 (4)	0.2040 (2)	0.0480 (13)	
H6	0.7599	0.5478	0.1979	0.058*	
N7	0.1980 (5)	0.5983 (5)	−0.0011 (2)	0.0640 (16)	
N8	0.7927 (6)	0.1075 (5)	0.3372 (3)	0.0712 (17)	
C1	0.2550 (6)	0.3818 (6)	0.2556 (3)	0.0525 (17)	
C2	0.2365 (7)	0.2816 (6)	0.2369 (3)	0.0622 (19)	
H2A	0.1693	0.2668	0.2536	0.075*	
C3	0.3138 (7)	0.2029 (6)	0.1944 (3)	0.0635 (19)	
C4	0.4164 (7)	0.2268 (6)	0.1695 (3)	0.0625 (19)	
H4A	0.4703	0.1753	0.1408	0.075*	
C5	0.4356 (7)	0.3237 (7)	0.1875 (3)	0.0614 (19)	
H5A	0.5033	0.3374	0.1706	0.074*	
C6	0.3577 (6)	0.4057 (6)	0.2310 (3)	0.0453 (15)	
C7	0.3832 (6)	0.5038 (6)	0.2507 (3)	0.0476 (16)	
H7A	0.4505	0.5176	0.2335	0.057*	
C8	0.3370 (6)	0.6713 (5)	0.3167 (3)	0.0427 (15)	
C9	0.4376 (6)	0.7001 (6)	0.2999 (3)	0.0509 (16)	
H9A	0.4946	0.6542	0.2705	0.061*	
C10	0.4569 (6)	0.7951 (6)	0.3252 (3)	0.0532 (17)	
C11	0.3742 (7)	0.8617 (6)	0.3700 (3)	0.0580 (18)	
C12	0.2731 (7)	0.8316 (6)	0.3877 (3)	0.0544 (17)	
H12A	0.2183	0.8757	0.4182	0.065*	
C13	0.2515 (6)	0.7399 (5)	0.3619 (3)	0.0458 (15)	
C14	0.1118 (6)	0.7099 (6)	0.4310 (3)	0.0535 (17)	
H14A	0.1613	0.7221	0.4609	0.064*	
C15	0.0009 (6)	0.6875 (5)	0.4493 (3)	0.0462 (15)	
C16	−0.0255 (6)	0.6752 (6)	0.5095 (3)	0.0548 (17)	
H16A	0.0279	0.6837	0.5386	0.066*	
C17	−0.1272 (6)	0.6510 (6)	0.5281 (3)	0.0567 (18)	
H17A	−0.1424	0.6447	0.5691	0.068*	
C18	−0.2091 (6)	0.6355 (6)	0.4851 (3)	0.0493 (16)	
C19	−0.1828 (6)	0.6490 (6)	0.4242 (3)	0.0534 (17)	
H19A	−0.2354	0.6397	0.3949	0.064*	
C20	−0.0815 (6)	0.6756 (5)	0.4062 (3)	0.0466 (15)	
C22	0.1668 (11)	0.0945 (8)	0.1939 (4)	0.104 (3)	
H22A	0.0974	0.1747	0.1995	0.124*	
H22B	0.1417	0.0532	0.1630	0.124*	
C23	0.1964 (13)	0.0245 (9)	0.2488 (5)	0.142 (5)	
H23A	0.1192	0.0230	0.2655	0.212*	
H23B	0.2325	0.0607	0.2769	0.212*	
H23C	0.2575	−0.0578	0.2413	0.212*	
C24	0.3764 (9)	0.0149 (7)	0.1341 (4)	0.092 (3)	
H24A	0.3718	−0.0625	0.1435	0.110*	
H24B	0.4645	0.0031	0.1407	0.110*	
C25	0.3448 (12)	0.0464 (9)	0.0695 (5)	0.125 (4)	
H25A	0.4029	−0.0186	0.0453	0.188*	
H25B	0.3530	0.1209	0.0590	0.188*	
H25C	0.2580	0.0575	0.0621	0.188*	
C26	0.5640 (8)	0.8280 (8)	0.3018 (4)	0.082 (2)	
H26A	0.6127	0.8353	0.3352	0.124*	
H26B	0.5273	0.9045	0.2794	0.124*	
H26C	0.6196	0.7650	0.2760	0.124*	
C27	0.3887 (9)	0.9674 (7)	0.3990 (4)	0.095 (3)	
H27A	0.4679	0.9371	0.4213	0.142*	
H27B	0.3178	1.0071	0.4258	0.142*	
H27C	0.3895	1.0250	0.3682	0.142*	
C29	−0.3988 (6)	0.6015 (8)	0.4570 (4)	0.078 (2)	
H29A	−0.4092	0.6620	0.4251	0.094*	
H29B	−0.4821	0.6232	0.4759	0.094*	
C30	−0.3571 (8)	0.4787 (9)	0.4297 (4)	0.098 (3)	
H30A	−0.4243	0.4790	0.4040	0.148*	
H30B	−0.3398	0.4172	0.4611	0.148*	
H30C	−0.2806	0.4611	0.4063	0.148*	
C31	−0.3373 (7)	0.5918 (6)	0.5643 (3)	0.065 (2)	
H31A	−0.2573	0.5568	0.5870	0.078*	
H31B	−0.3755	0.5335	0.5667	0.078*	
C32	−0.4263 (9)	0.7071 (8)	0.5938 (4)	0.102 (3)	
H32A	−0.4430	0.6883	0.6345	0.153*	
H32B	−0.5058	0.7431	0.5717	0.153*	
H32C	−0.3872	0.7638	0.5941	0.153*	
C33	0.4194 (6)	0.6733 (6)	0.0906 (3)	0.0504 (16)	
C34	0.3201 (6)	0.6427 (6)	0.0748 (3)	0.0551 (17)	
H34A	0.2691	0.6304	0.1049	0.066*	
C35	0.2952 (6)	0.6301 (6)	0.0143 (3)	0.0521 (17)	
C36	0.3750 (7)	0.6504 (6)	−0.0296 (3)	0.0614 (19)	
H36A	0.3602	0.6441	−0.0702	0.074*	
C37	0.4733 (6)	0.6790 (6)	−0.0132 (3)	0.0570 (18)	
H37A	0.5245	0.6912	−0.0432	0.068*	
C38	0.4996 (6)	0.6905 (5)	0.0467 (3)	0.0467 (15)	
C39	0.6070 (6)	0.7153 (6)	0.0633 (3)	0.0558 (17)	
H39A	0.6556	0.7292	0.0326	0.067*	
C40	0.7476 (6)	0.7447 (6)	0.1312 (3)	0.0495 (16)	
C41	0.7670 (7)	0.8394 (7)	0.1032 (3)	0.068 (2)	
H41A	0.7097	0.8854	0.0731	0.081*	
C42	0.8680 (8)	0.8700 (7)	0.1176 (4)	0.067 (2)	
C43	0.9524 (7)	0.8001 (7)	0.1628 (4)	0.066 (2)	
C44	0.9355 (6)	0.7039 (6)	0.1913 (3)	0.0554 (17)	
H44A	0.9940	0.6567	0.2208	0.066*	
C45	0.8327 (6)	0.6764 (6)	0.1768 (3)	0.0481 (16)	
C46	0.8779 (6)	0.5096 (6)	0.2494 (3)	0.0466 (15)	
H46A	0.9423	0.5257	0.2671	0.056*	
C47	0.8537 (5)	0.4122 (6)	0.2719 (3)	0.0437 (15)	
C48	0.9273 (6)	0.3351 (6)	0.3189 (3)	0.0546 (17)	
H48A	0.9916	0.3523	0.3360	0.065*	
C49	0.9098 (7)	0.2369 (6)	0.3407 (3)	0.0614 (19)	
H49A	0.9620	0.1887	0.3719	0.074*	
C50	0.8121 (6)	0.2061 (6)	0.3164 (3)	0.0522 (16)	
C51	0.7368 (6)	0.2830 (6)	0.2700 (3)	0.0536 (17)	
H51A	0.6723	0.2655	0.2533	0.064*	
C52	0.7536 (6)	0.3841 (6)	0.2475 (3)	0.0466 (15)	
C54	0.1720 (7)	0.5812 (6)	−0.0635 (3)	0.066 (2)	
H54A	0.1355	0.5216	−0.0643	0.079*	
H54B	0.2525	0.5484	−0.0855	0.079*	
C55	0.0812 (9)	0.6977 (8)	−0.0946 (4)	0.101 (3)	
H55A	0.0584	0.6796	−0.1334	0.151*	
H55B	0.1223	0.7526	−0.0996	0.151*	
H55C	0.0050	0.7356	−0.0707	0.151*	
C56	0.1057 (7)	0.5896 (8)	0.0441 (4)	0.076 (2)	
H56A	0.0239	0.6093	0.0242	0.091*	
H56B	0.0916	0.6504	0.0744	0.091*	
C57	0.1492 (8)	0.4652 (9)	0.0751 (4)	0.094 (3)	
H57A	0.0862	0.4668	0.1044	0.140*	
H57B	0.2302	0.4446	0.0948	0.140*	
H57C	0.1589	0.4049	0.0457	0.140*	
C58	0.8813 (11)	0.9777 (8)	0.0858 (5)	0.120 (4)	
H58A	0.8859	1.0326	0.1153	0.180*	
H58B	0.8080	1.0202	0.0601	0.180*	
H58C	0.9583	0.9484	0.0620	0.180*	
C59	1.0633 (8)	0.8279 (8)	0.1837 (4)	0.091 (3)	
H59A	1.1080	0.8407	0.1492	0.137*	
H59B	1.1216	0.7601	0.2079	0.137*	
H59C	1.0302	0.9003	0.2072	0.137*	
C61	0.8788 (9)	0.0184 (7)	0.3812 (4)	0.085 (3)	
H61A	0.9642	0.0163	0.3772	0.102*	
H61B	0.8849	−0.0624	0.3726	0.102*	
C62	0.8333 (11)	0.0484 (9)	0.4446 (4)	0.123 (4)	
H62A	0.8955	−0.0087	0.4720	0.184*	
H62B	0.7524	0.0432	0.4498	0.184*	
H62C	0.8228	0.1300	0.4527	0.184*	
C63	0.6915 (8)	0.0789 (7)	0.3124 (3)	0.076 (2)	
H63A	0.6165	0.1544	0.3032	0.092*	
H63B	0.6666	0.0321	0.3421	0.092*	
C64	0.7357 (11)	0.0071 (8)	0.2564 (4)	0.110 (3)	
H64A	0.6669	−0.0090	0.2404	0.165*	
H64B	0.8079	−0.0691	0.2658	0.165*	
H64C	0.7609	0.0532	0.2270	0.165*	

### Atomic displacement parameters (Å^2^)

**Table:**

	*U*^11^	*U*^22^	*U*^33^	*U*^12^	*U*^13^	*U*^23^
O1	0.055 (3)	0.077 (3)	0.085 (4)	−0.041 (3)	0.024 (3)	−0.035 (3)
O2	0.059 (3)	0.087 (3)	0.051 (3)	−0.041 (3)	0.000 (2)	−0.004 (2)
O3	0.061 (3)	0.091 (4)	0.056 (3)	−0.042 (3)	−0.002 (2)	−0.009 (3)
O4	0.051 (3)	0.076 (3)	0.069 (3)	−0.035 (3)	−0.020 (2)	0.018 (3)
N1	0.045 (3)	0.049 (3)	0.045 (3)	−0.025 (3)	0.002 (2)	−0.009 (3)
N2	0.047 (3)	0.048 (3)	0.052 (3)	−0.025 (3)	0.005 (3)	−0.011 (3)
N3	0.100 (6)	0.078 (5)	0.116 (6)	−0.058 (4)	0.027 (4)	−0.043 (4)
N4	0.043 (3)	0.083 (4)	0.061 (4)	−0.031 (3)	0.001 (3)	0.008 (3)
N5	0.044 (3)	0.046 (3)	0.067 (4)	−0.022 (3)	0.000 (3)	−0.003 (3)
N6	0.048 (3)	0.045 (3)	0.059 (4)	−0.028 (3)	0.001 (3)	−0.007 (3)
N7	0.049 (3)	0.084 (4)	0.060 (4)	−0.028 (3)	−0.004 (3)	−0.015 (3)
N8	0.080 (4)	0.049 (4)	0.094 (5)	−0.037 (3)	0.000 (4)	0.007 (3)
C1	0.047 (4)	0.053 (4)	0.060 (4)	−0.023 (3)	0.005 (3)	−0.018 (3)
C2	0.066 (5)	0.058 (5)	0.075 (5)	−0.039 (4)	0.018 (4)	−0.018 (4)
C3	0.066 (5)	0.055 (4)	0.078 (5)	−0.033 (4)	0.001 (4)	−0.016 (4)
C4	0.054 (4)	0.065 (5)	0.066 (5)	−0.021 (4)	0.012 (4)	−0.025 (4)
C5	0.052 (4)	0.077 (5)	0.062 (5)	−0.033 (4)	0.013 (4)	−0.019 (4)
C6	0.036 (3)	0.052 (4)	0.049 (4)	−0.020 (3)	0.001 (3)	−0.006 (3)
C7	0.047 (4)	0.058 (4)	0.047 (4)	−0.030 (3)	−0.003 (3)	0.002 (3)
C8	0.041 (4)	0.043 (4)	0.052 (4)	−0.025 (3)	−0.004 (3)	0.002 (3)
C9	0.050 (4)	0.047 (4)	0.065 (4)	−0.030 (3)	0.002 (3)	−0.004 (3)
C10	0.049 (4)	0.058 (4)	0.063 (4)	−0.034 (3)	−0.003 (3)	0.004 (4)
C11	0.070 (5)	0.049 (4)	0.069 (5)	−0.038 (4)	−0.011 (4)	−0.001 (4)
C12	0.066 (5)	0.046 (4)	0.060 (4)	−0.032 (4)	0.008 (3)	−0.007 (3)
C13	0.047 (4)	0.043 (4)	0.054 (4)	−0.025 (3)	−0.007 (3)	−0.001 (3)
C14	0.041 (4)	0.047 (4)	0.069 (5)	−0.015 (3)	−0.010 (3)	0.000 (3)
C15	0.041 (4)	0.042 (4)	0.053 (4)	−0.015 (3)	0.000 (3)	−0.007 (3)
C16	0.054 (4)	0.061 (4)	0.053 (4)	−0.028 (4)	−0.005 (3)	−0.004 (3)
C17	0.057 (4)	0.067 (5)	0.050 (4)	−0.029 (4)	0.006 (3)	−0.005 (3)
C18	0.042 (4)	0.048 (4)	0.054 (4)	−0.016 (3)	0.003 (3)	0.003 (3)
C19	0.042 (4)	0.060 (4)	0.062 (5)	−0.025 (3)	−0.005 (3)	−0.001 (3)
C20	0.043 (4)	0.050 (4)	0.045 (4)	−0.017 (3)	0.003 (3)	−0.006 (3)
C22	0.173 (11)	0.065 (6)	0.081 (6)	−0.057 (6)	−0.044 (7)	0.002 (5)
C23	0.198 (13)	0.096 (8)	0.131 (10)	−0.062 (8)	−0.073 (9)	0.011 (7)
C24	0.117 (8)	0.057 (5)	0.106 (7)	−0.040 (5)	0.003 (6)	−0.027 (5)
C25	0.164 (11)	0.097 (8)	0.109 (9)	−0.047 (7)	−0.002 (8)	−0.022 (6)
C26	0.073 (5)	0.088 (6)	0.113 (7)	−0.060 (5)	0.005 (5)	−0.012 (5)
C27	0.111 (7)	0.080 (6)	0.122 (7)	−0.069 (6)	0.006 (6)	−0.024 (5)
C29	0.033 (4)	0.120 (7)	0.081 (6)	−0.033 (4)	−0.005 (4)	0.023 (5)
C30	0.075 (6)	0.134 (9)	0.110 (7)	−0.066 (6)	−0.003 (5)	−0.018 (6)
C31	0.056 (4)	0.063 (5)	0.070 (5)	−0.018 (4)	0.018 (4)	−0.002 (4)
C32	0.084 (6)	0.089 (7)	0.111 (7)	−0.015 (5)	0.027 (5)	−0.011 (5)
C33	0.045 (4)	0.054 (4)	0.049 (4)	−0.016 (3)	−0.004 (3)	−0.008 (3)
C34	0.045 (4)	0.063 (4)	0.057 (4)	−0.022 (3)	0.007 (3)	−0.009 (3)
C35	0.043 (4)	0.053 (4)	0.057 (4)	−0.017 (3)	−0.006 (3)	−0.005 (3)
C36	0.059 (5)	0.070 (5)	0.052 (4)	−0.024 (4)	−0.008 (4)	−0.003 (4)
C37	0.051 (4)	0.065 (5)	0.053 (4)	−0.022 (4)	−0.001 (3)	0.006 (3)
C38	0.042 (4)	0.038 (4)	0.057 (4)	−0.014 (3)	−0.003 (3)	−0.002 (3)
C39	0.058 (4)	0.048 (4)	0.058 (5)	−0.020 (3)	0.002 (4)	−0.004 (3)
C40	0.052 (4)	0.043 (4)	0.059 (4)	−0.026 (3)	0.008 (3)	−0.013 (3)
C41	0.071 (5)	0.061 (5)	0.078 (5)	−0.034 (4)	0.008 (4)	−0.002 (4)
C42	0.082 (6)	0.060 (5)	0.080 (5)	−0.050 (4)	0.011 (4)	−0.008 (4)
C43	0.066 (5)	0.052 (4)	0.097 (6)	−0.041 (4)	0.015 (4)	−0.021 (4)
C44	0.055 (4)	0.057 (4)	0.062 (4)	−0.030 (4)	0.007 (3)	−0.015 (3)
C45	0.042 (4)	0.043 (4)	0.067 (4)	−0.026 (3)	0.007 (3)	−0.009 (3)
C46	0.044 (4)	0.054 (4)	0.050 (4)	−0.028 (3)	−0.002 (3)	−0.009 (3)
C47	0.030 (3)	0.051 (4)	0.052 (4)	−0.020 (3)	−0.001 (3)	−0.007 (3)
C48	0.051 (4)	0.066 (5)	0.055 (4)	−0.031 (4)	−0.008 (3)	−0.007 (4)
C49	0.054 (4)	0.060 (5)	0.072 (5)	−0.027 (4)	−0.009 (4)	0.004 (4)
C50	0.052 (4)	0.051 (4)	0.057 (4)	−0.025 (3)	0.007 (3)	−0.006 (3)
C51	0.047 (4)	0.048 (4)	0.068 (5)	−0.022 (3)	−0.010 (3)	0.002 (3)
C52	0.038 (4)	0.051 (4)	0.050 (4)	−0.018 (3)	−0.001 (3)	−0.002 (3)
C54	0.057 (4)	0.070 (5)	0.063 (5)	−0.017 (4)	−0.019 (4)	−0.007 (4)
C55	0.095 (7)	0.083 (6)	0.101 (7)	−0.014 (5)	−0.031 (5)	0.012 (5)
C56	0.040 (4)	0.105 (7)	0.086 (6)	−0.031 (4)	0.003 (4)	−0.028 (5)
C57	0.072 (6)	0.120 (8)	0.096 (7)	−0.048 (6)	0.005 (5)	−0.005 (6)
C58	0.144 (10)	0.081 (7)	0.164 (10)	−0.076 (7)	0.006 (8)	0.011 (6)
C59	0.082 (6)	0.087 (6)	0.134 (8)	−0.064 (5)	0.011 (5)	−0.024 (5)
C61	0.102 (7)	0.052 (5)	0.100 (7)	−0.030 (5)	−0.008 (5)	0.009 (5)
C62	0.163 (11)	0.105 (8)	0.085 (7)	−0.043 (7)	−0.029 (7)	0.021 (6)
C63	0.111 (7)	0.074 (5)	0.072 (5)	−0.066 (5)	0.024 (5)	−0.009 (4)
C64	0.153 (10)	0.084 (6)	0.108 (8)	−0.062 (7)	0.038 (7)	−0.030 (6)

### Geometric parameters (Å, °)

**Table:**

O1—C1	1.326 (7)	C27—H27B	0.9600
O1—H1	0.9059	C27—H27C	0.9600
O2—C20	1.346 (7)	C29—C30	1.495 (11)
O2—H2	0.9029	C29—H29A	0.9700
O3—C33	1.361 (7)	C29—H29B	0.9700
O3—H3	0.8998	C30—H30A	0.9600
O4—C52	1.319 (7)	C30—H30B	0.9600
N1—C7	1.309 (7)	C30—H30C	0.9600
N1—C8	1.407 (7)	C31—C32	1.498 (10)
N2—C14	1.283 (8)	C31—H31A	0.9700
N2—C13	1.411 (7)	C31—H31B	0.9700
N3—C3	1.359 (8)	C32—H32A	0.9600
N3—C24	1.459 (10)	C32—H32B	0.9600
N3—C22	1.556 (12)	C32—H32C	0.9600
N4—C18	1.363 (8)	C33—C34	1.384 (9)
N4—C31	1.435 (8)	C33—C38	1.401 (9)
N4—C29	1.475 (9)	C34—C35	1.401 (9)
N5—C39	1.287 (8)	C34—H34A	0.9300
N5—C40	1.423 (8)	C35—C36	1.411 (9)
N6—C46	1.307 (7)	C36—C37	1.363 (9)
N6—C45	1.397 (7)	C36—H36A	0.9300
N6—H6	0.8542	C37—C38	1.388 (8)
N7—C35	1.368 (8)	C37—H37A	0.9300
N7—C54	1.455 (8)	C38—C39	1.434 (9)
N7—C56	1.481 (9)	C39—H39A	0.9300
N8—C50	1.362 (8)	C40—C41	1.378 (9)
N8—C63	1.458 (9)	C40—C45	1.394 (9)
N8—C61	1.463 (10)	C41—C42	1.394 (10)
C1—C2	1.384 (8)	C41—H41A	0.9300
C1—C6	1.422 (8)	C42—C43	1.390 (10)
C2—C3	1.379 (9)	C42—C58	1.517 (10)
C2—H2A	0.9300	C43—C44	1.384 (9)
C3—C4	1.422 (9)	C43—C59	1.521 (10)
C4—C5	1.346 (9)	C44—C45	1.393 (8)
C4—H4A	0.9300	C44—H44A	0.9300
C5—C6	1.413 (8)	C46—C47	1.390 (8)
C5—H5A	0.9300	C46—H46A	0.9300
C6—C7	1.410 (8)	C47—C48	1.400 (8)
C7—H7A	0.9300	C47—C52	1.438 (8)
C8—C9	1.379 (8)	C48—C49	1.353 (9)
C8—C13	1.413 (8)	C48—H48A	0.9300
C9—C10	1.385 (8)	C49—C50	1.432 (9)
C9—H9A	0.9300	C49—H49A	0.9300
C10—C11	1.385 (9)	C50—C51	1.397 (9)
C10—C26	1.519 (9)	C51—C52	1.386 (8)
C11—C12	1.398 (9)	C51—H51A	0.9300
C11—C27	1.511 (9)	C54—C55	1.501 (10)
C12—C13	1.369 (8)	C54—H54A	0.9700
C12—H12A	0.9300	C54—H54B	0.9700
C14—C15	1.455 (9)	C55—H55A	0.9600
C14—H14A	0.9300	C55—H55B	0.9600
C15—C16	1.382 (8)	C55—H55C	0.9600
C15—C20	1.402 (8)	C56—C57	1.509 (11)
C16—C17	1.369 (9)	C56—H56A	0.9700
C16—H16A	0.9300	C56—H56B	0.9700
C17—C18	1.412 (9)	C57—H57A	0.9600
C17—H17A	0.9300	C57—H57B	0.9600
C18—C19	1.400 (9)	C57—H57C	0.9600
C19—C20	1.378 (8)	C58—H58A	0.9600
C19—H19A	0.9300	C58—H58B	0.9600
C22—C23	1.420 (12)	C58—H58C	0.9600
C22—H22A	0.9700	C59—H59A	0.9600
C22—H22B	0.9700	C59—H59B	0.9600
C23—H23A	0.9600	C59—H59C	0.9600
C23—H23B	0.9600	C61—C62	1.495 (12)
C23—H23C	0.9600	C61—H61A	0.9700
C24—C25	1.480 (12)	C61—H61B	0.9700
C24—H24A	0.9700	C62—H62A	0.9600
C24—H24B	0.9700	C62—H62B	0.9600
C25—H25A	0.9600	C62—H62C	0.9600
C25—H25B	0.9600	C63—C64	1.492 (10)
C25—H25C	0.9600	C63—H63A	0.9700
C26—H26A	0.9600	C63—H63B	0.9700
C26—H26B	0.9600	C64—H64A	0.9600
C26—H26C	0.9600	C64—H64B	0.9600
C27—H27A	0.9600	C64—H64C	0.9600
			
C1—O1—H1	99.1	C32—C31—H31A	108.5
C20—O2—H2	107.2	N4—C31—H31B	108.5
C33—O3—H3	111.0	C32—C31—H31B	108.5
C7—N1—C8	123.9 (5)	H31A—C31—H31B	107.5
C14—N2—C13	119.7 (5)	C31—C32—H32A	109.5
C3—N3—C24	124.4 (7)	C31—C32—H32B	109.5
C3—N3—C22	119.8 (7)	H32A—C32—H32B	109.5
C24—N3—C22	115.5 (6)	C31—C32—H32C	109.5
C18—N4—C31	122.9 (6)	H32A—C32—H32C	109.5
C18—N4—C29	120.4 (6)	H32B—C32—H32C	109.5
C31—N4—C29	116.5 (6)	O3—C33—C34	117.0 (6)
C39—N5—C40	120.7 (6)	O3—C33—C38	121.9 (6)
C46—N6—C45	126.5 (5)	C34—C33—C38	121.0 (6)
C46—N6—H6	99.8	C33—C34—C35	121.1 (6)
C45—N6—H6	133.7	C33—C34—H34A	119.4
C35—N7—C54	122.2 (6)	C35—C34—H34A	119.4
C35—N7—C56	121.9 (6)	N7—C35—C34	121.0 (6)
C54—N7—C56	115.7 (6)	N7—C35—C36	121.8 (6)
C50—N8—C63	121.2 (6)	C34—C35—C36	117.2 (6)
C50—N8—C61	122.3 (6)	C37—C36—C35	121.0 (6)
C63—N8—C61	116.2 (6)	C37—C36—H36A	119.5
O1—C1—C2	119.1 (6)	C35—C36—H36A	119.5
O1—C1—C6	121.1 (5)	C36—C37—C38	122.3 (6)
C2—C1—C6	119.8 (6)	C36—C37—H37A	118.9
C3—C2—C1	122.5 (6)	C38—C37—H37A	118.9
C3—C2—H2A	118.7	C37—C38—C33	117.4 (6)
C1—C2—H2A	118.7	C37—C38—C39	121.6 (6)
N3—C3—C2	121.8 (7)	C33—C38—C39	121.0 (6)
N3—C3—C4	120.4 (7)	N5—C39—C38	123.9 (6)
C2—C3—C4	117.9 (6)	N5—C39—H39A	118.1
C5—C4—C3	120.2 (6)	C38—C39—H39A	118.1
C5—C4—H4A	119.9	C41—C40—C45	118.3 (6)
C3—C4—H4A	119.9	C41—C40—N5	122.6 (6)
C4—C5—C6	123.0 (6)	C45—C40—N5	119.0 (6)
C4—C5—H5A	118.5	C40—C41—C42	123.5 (7)
C6—C5—H5A	118.5	C40—C41—H41A	118.2
C7—C6—C5	121.8 (6)	C42—C41—H41A	118.2
C7—C6—C1	121.5 (6)	C43—C42—C41	117.2 (7)
C5—C6—C1	116.6 (6)	C43—C42—C58	122.2 (7)
N1—C7—C6	120.9 (6)	C41—C42—C58	120.5 (8)
N1—C7—H7A	119.5	C44—C43—C42	120.3 (6)
C6—C7—H7A	119.5	C44—C43—C59	117.8 (8)
C9—C8—N1	124.8 (6)	C42—C43—C59	121.8 (7)
C9—C8—C13	118.8 (5)	C43—C44—C45	121.3 (7)
N1—C8—C13	116.3 (5)	C43—C44—H44A	119.4
C8—C9—C10	122.4 (6)	C45—C44—H44A	119.4
C8—C9—H9A	118.8	C44—C45—C40	119.2 (6)
C10—C9—H9A	118.8	C44—C45—N6	123.0 (6)
C9—C10—C11	119.0 (6)	C40—C45—N6	117.7 (5)
C9—C10—C26	119.9 (6)	N6—C46—C47	122.0 (5)
C11—C10—C26	121.0 (6)	N6—C46—H46A	119.0
C10—C11—C12	118.7 (6)	C47—C46—H46A	119.0
C10—C11—C27	121.7 (7)	C46—C47—C48	121.5 (5)
C12—C11—C27	119.5 (7)	C46—C47—C52	121.8 (6)
C13—C12—C11	122.6 (6)	C48—C47—C52	116.7 (6)
C13—C12—H12A	118.7	C49—C48—C47	123.3 (6)
C11—C12—H12A	118.7	C49—C48—H48A	118.3
C12—C13—N2	123.7 (6)	C47—C48—H48A	118.3
C12—C13—C8	118.4 (6)	C48—C49—C50	120.9 (6)
N2—C13—C8	117.8 (5)	C48—C49—H49A	119.5
N2—C14—C15	123.4 (6)	C50—C49—H49A	119.5
N2—C14—H14A	118.3	N8—C50—C51	121.3 (6)
C15—C14—H14A	118.3	N8—C50—C49	122.2 (6)
C16—C15—C20	117.9 (6)	C51—C50—C49	116.5 (6)
C16—C15—C14	121.3 (6)	C52—C51—C50	123.0 (6)
C20—C15—C14	120.8 (6)	C52—C51—H51A	118.5
C17—C16—C15	122.6 (6)	C50—C51—H51A	118.5
C17—C16—H16A	118.7	O4—C52—C51	120.3 (6)
C15—C16—H16A	118.7	O4—C52—C47	120.1 (6)
C16—C17—C18	120.2 (6)	C51—C52—C47	119.6 (6)
C16—C17—H17A	119.9	N7—C54—C55	113.0 (6)
C18—C17—H17A	119.9	N7—C54—H54A	109.0
N4—C18—C19	121.5 (6)	C55—C54—H54A	109.0
N4—C18—C17	121.4 (6)	N7—C54—H54B	109.0
C19—C18—C17	117.1 (6)	C55—C54—H54B	109.0
C20—C19—C18	122.1 (6)	H54A—C54—H54B	107.8
C20—C19—H19A	119.0	C54—C55—H55A	109.5
C18—C19—H19A	119.0	C54—C55—H55B	109.5
O2—C20—C19	118.6 (5)	H55A—C55—H55B	109.5
O2—C20—C15	121.3 (6)	C54—C55—H55C	109.5
C19—C20—C15	120.1 (6)	H55A—C55—H55C	109.5
C23—C22—N3	104.9 (9)	H55B—C55—H55C	109.5
C23—C22—H22A	110.8	N7—C56—C57	114.0 (6)
N3—C22—H22A	110.8	N7—C56—H56A	108.8
C23—C22—H22B	110.8	C57—C56—H56A	108.8
N3—C22—H22B	110.8	N7—C56—H56B	108.8
H22A—C22—H22B	108.8	C57—C56—H56B	108.8
C22—C23—H23A	109.5	H56A—C56—H56B	107.7
C22—C23—H23B	109.5	C56—C57—H57A	109.5
H23A—C23—H23B	109.5	C56—C57—H57B	109.5
C22—C23—H23C	109.5	H57A—C57—H57B	109.5
H23A—C23—H23C	109.5	C56—C57—H57C	109.5
H23B—C23—H23C	109.5	H57A—C57—H57C	109.5
N3—C24—C25	115.0 (8)	H57B—C57—H57C	109.5
N3—C24—H24A	108.5	C42—C58—H58A	109.5
C25—C24—H24A	108.5	C42—C58—H58B	109.5
N3—C24—H24B	108.5	H58A—C58—H58B	109.5
C25—C24—H24B	108.5	C42—C58—H58C	109.5
H24A—C24—H24B	107.5	H58A—C58—H58C	109.5
C24—C25—H25A	109.5	H58B—C58—H58C	109.5
C24—C25—H25B	109.5	C43—C59—H59A	109.5
H25A—C25—H25B	109.5	C43—C59—H59B	109.5
C24—C25—H25C	109.5	H59A—C59—H59B	109.5
H25A—C25—H25C	109.5	C43—C59—H59C	109.5
H25B—C25—H25C	109.5	H59A—C59—H59C	109.5
C10—C26—H26A	109.5	H59B—C59—H59C	109.5
C10—C26—H26B	109.5	N8—C61—C62	112.3 (7)
H26A—C26—H26B	109.5	N8—C61—H61A	109.1
C10—C26—H26C	109.5	C62—C61—H61A	109.1
H26A—C26—H26C	109.5	N8—C61—H61B	109.1
H26B—C26—H26C	109.5	C62—C61—H61B	109.1
C11—C27—H27A	109.5	H61A—C61—H61B	107.9
C11—C27—H27B	109.5	C61—C62—H62A	109.5
H27A—C27—H27B	109.5	C61—C62—H62B	109.5
C11—C27—H27C	109.5	H62A—C62—H62B	109.5
H27A—C27—H27C	109.5	C61—C62—H62C	109.5
H27B—C27—H27C	109.5	H62A—C62—H62C	109.5
N4—C29—C30	113.8 (6)	H62B—C62—H62C	109.5
N4—C29—H29A	108.8	N8—C63—C64	111.0 (7)
C30—C29—H29A	108.8	N8—C63—H63A	109.4
N4—C29—H29B	108.8	C64—C63—H63A	109.4
C30—C29—H29B	108.8	N8—C63—H63B	109.4
H29A—C29—H29B	107.7	C64—C63—H63B	109.4
C29—C30—H30A	109.5	H63A—C63—H63B	108.0
C29—C30—H30B	109.5	C63—C64—H64A	109.5
H30A—C30—H30B	109.5	C63—C64—H64B	109.5
C29—C30—H30C	109.5	H64A—C64—H64B	109.5
H30A—C30—H30C	109.5	C63—C64—H64C	109.5
H30B—C30—H30C	109.5	H64A—C64—H64C	109.5
N4—C31—C32	114.9 (6)	H64B—C64—H64C	109.5
N4—C31—H31A	108.5		
			
O1—C1—C2—C3	−179.7 (7)	O3—C33—C34—C35	−179.8 (6)
C6—C1—C2—C3	−0.5 (11)	C38—C33—C34—C35	−1.1 (10)
C24—N3—C3—C2	−175.9 (8)	C54—N7—C35—C34	−178.3 (6)
C22—N3—C3—C2	11.0 (12)	C56—N7—C35—C34	7.1 (10)
C24—N3—C3—C4	4.8 (13)	C54—N7—C35—C36	1.1 (10)
C22—N3—C3—C4	−168.4 (7)	C56—N7—C35—C36	−173.5 (7)
C1—C2—C3—N3	−179.1 (7)	C33—C34—C35—N7	179.1 (6)
C1—C2—C3—C4	0.3 (11)	C33—C34—C35—C36	−0.3 (9)
N3—C3—C4—C5	179.3 (7)	N7—C35—C36—C37	−178.4 (6)
C2—C3—C4—C5	−0.1 (11)	C34—C35—C36—C37	1.1 (10)
C3—C4—C5—C6	0.0 (11)	C35—C36—C37—C38	−0.5 (10)
C4—C5—C6—C7	177.5 (6)	C36—C37—C38—C33	−0.9 (10)
C4—C5—C6—C1	−0.3 (10)	C36—C37—C38—C39	177.2 (6)
O1—C1—C6—C7	1.9 (10)	O3—C33—C38—C37	−179.7 (6)
C2—C1—C6—C7	−177.3 (6)	C34—C33—C38—C37	1.7 (9)
O1—C1—C6—C5	179.6 (6)	O3—C33—C38—C39	2.2 (9)
C2—C1—C6—C5	0.5 (9)	C34—C33—C38—C39	−176.4 (6)
C8—N1—C7—C6	176.9 (5)	C40—N5—C39—C38	−179.9 (6)
C5—C6—C7—N1	−177.0 (6)	C37—C38—C39—N5	−175.6 (6)
C1—C6—C7—N1	0.6 (9)	C33—C38—C39—N5	2.4 (10)
C7—N1—C8—C9	−1.5 (9)	C39—N5—C40—C41	47.0 (9)
C7—N1—C8—C13	179.8 (6)	C39—N5—C40—C45	−137.3 (6)
N1—C8—C9—C10	−179.5 (6)	C45—C40—C41—C42	0.6 (10)
C13—C8—C9—C10	−0.9 (9)	N5—C40—C41—C42	176.4 (6)
C8—C9—C10—C11	1.7 (10)	C40—C41—C42—C43	0.0 (11)
C8—C9—C10—C26	−175.8 (6)	C40—C41—C42—C58	−178.5 (7)
C9—C10—C11—C12	−0.8 (10)	C41—C42—C43—C44	0.4 (11)
C26—C10—C11—C12	176.7 (6)	C58—C42—C43—C44	178.9 (7)
C9—C10—C11—C27	−179.2 (7)	C41—C42—C43—C59	−177.9 (7)
C26—C10—C11—C27	−1.8 (11)	C58—C42—C43—C59	0.6 (12)
C10—C11—C12—C13	−0.9 (10)	C42—C43—C44—C45	−1.5 (10)
C27—C11—C12—C13	177.6 (7)	C59—C43—C44—C45	176.9 (6)
C11—C12—C13—N2	−175.2 (6)	C43—C44—C45—C40	2.1 (9)
C11—C12—C13—C8	1.7 (10)	C43—C44—C45—N6	179.2 (6)
C14—N2—C13—C12	−44.7 (9)	C41—C40—C45—C44	−1.6 (9)
C14—N2—C13—C8	138.4 (6)	N5—C40—C45—C44	−177.6 (5)
C9—C8—C13—C12	−0.8 (9)	C41—C40—C45—N6	−178.8 (6)
N1—C8—C13—C12	178.0 (5)	N5—C40—C45—N6	5.2 (8)
C9—C8—C13—N2	176.3 (5)	C46—N6—C45—C44	7.0 (9)
N1—C8—C13—N2	−4.9 (8)	C46—N6—C45—C40	−175.9 (6)
C13—N2—C14—C15	177.9 (5)	C45—N6—C46—C47	−177.8 (6)
N2—C14—C15—C16	175.1 (6)	N6—C46—C47—C48	177.3 (6)
N2—C14—C15—C20	−3.7 (9)	N6—C46—C47—C52	−1.5 (9)
C20—C15—C16—C17	0.6 (10)	C46—C47—C48—C49	−177.6 (6)
C14—C15—C16—C17	−178.3 (6)	C52—C47—C48—C49	1.3 (10)
C15—C16—C17—C18	1.0 (10)	C47—C48—C49—C50	−0.3 (11)
C31—N4—C18—C19	179.6 (6)	C63—N8—C50—C51	−0.8 (10)
C29—N4—C18—C19	−6.1 (10)	C61—N8—C50—C51	173.3 (7)
C31—N4—C18—C17	0.6 (10)	C63—N8—C50—C49	179.1 (6)
C29—N4—C18—C17	174.9 (6)	C61—N8—C50—C49	−6.7 (10)
C16—C17—C18—N4	177.6 (6)	C48—C49—C50—N8	179.6 (6)
C16—C17—C18—C19	−1.5 (9)	C48—C49—C50—C51	−0.4 (10)
N4—C18—C19—C20	−178.8 (6)	N8—C50—C51—C52	−179.9 (6)
C17—C18—C19—C20	0.3 (9)	C49—C50—C51—C52	0.1 (10)
C18—C19—C20—O2	−179.7 (6)	C50—C51—C52—O4	179.0 (6)
C18—C19—C20—C15	1.4 (10)	C50—C51—C52—C47	0.9 (10)
C16—C15—C20—O2	179.3 (6)	C46—C47—C52—O4	−0.8 (9)
C14—C15—C20—O2	−1.8 (9)	C48—C47—C52—O4	−179.7 (6)
C16—C15—C20—C19	−1.8 (9)	C46—C47—C52—C51	177.4 (6)
C14—C15—C20—C19	177.1 (6)	C48—C47—C52—C51	−1.5 (9)
C3—N3—C22—C23	−91.3 (9)	C35—N7—C54—C55	−88.1 (9)
C24—N3—C22—C23	94.9 (9)	C56—N7—C54—C55	86.8 (9)
C3—N3—C24—C25	−87.8 (11)	C35—N7—C56—C57	−88.4 (8)
C22—N3—C24—C25	85.7 (10)	C54—N7—C56—C57	96.7 (7)
C18—N4—C29—C30	88.3 (8)	C50—N8—C61—C62	93.5 (9)
C31—N4—C29—C30	−97.1 (8)	C63—N8—C61—C62	−92.1 (9)
C18—N4—C31—C32	88.0 (9)	C50—N8—C63—C64	83.9 (9)
C29—N4—C31—C32	−86.5 (9)	C61—N8—C63—C64	−90.6 (8)

### Hydrogen-bond geometry (Å, °)

**Table:**

*D*—H···*A*	*D*—H	H···*A*	*D*···*A*	*D*—H···*A*
O1—H1···N1	0.91	1.68	2.553 (7)	160
O2—H2···N2	0.90	1.79	2.613 (8)	150
O3—H3···N5	0.90	1.85	2.627 (8)	143
N6—H6···O4	0.85	1.74	2.562 (7)	162
C7—H7A···O4	0.93	2.50	3.360 (9)	153
C46—H46A···O1^i^	0.93	2.54	3.373 (9)	149

Symmetry codes: (i) *x*+1, *y*, *z*.
